# Proprietary arabinogalactan extract increases antibody response to the pneumonia vaccine: a randomized, double-blind, placebo-controlled, pilot study in healthy volunteers

**DOI:** 10.1186/1475-2891-9-32

**Published:** 2010-08-26

**Authors:** Jay K Udani, Betsy B Singh, Marilyn L Barrett, Vijay J Singh

**Affiliations:** 1 Medicus Research LLC, Northridge, CA 91325, USA; 2 UCLA School of Medicine, Department of Medicine, Los Angeles, CA 90024, USA; 3 Pharmacognosy Consulting, Mill Valley, CA 94941, USA

## Abstract

**Background:**

Arabinogalactan from Larch tree (*Larix *spp.) bark has previously demonstrated immunostimulatory activity. The purpose of this study was to test the hypothesis that ingestion of a proprietary arabinogalactan extract, ResistAid™, would selectively enhance the antibody response to the pneumococcal (pneumonia) vaccine in healthy adults.

**Methods:**

This randomized, double-blind, placebo-controlled, parallel group pilot study included 45 healthy adults who had not previously been vaccinated against *Streptococcus pneumoniae*. The volunteers began taking the study product or placebo (daily dosage 4.5 g) at the screening visit (V1-Day 0) and continued over the entire 72 day study period. After 30 days the subjects received the 23-valent pneumococcal vaccine (V2). They were monitored the following day (V3-Day 31), as well as 21 days (V4-Day 51) and 42 days (V5-Day 72) after vaccination. Responses by the adaptive immune system (antigen specific) were measured via pneumococcal IgG antibodies (subtypes 4, 6B, 9V, 14, 18C, 19F, and 23F) and salivary IgA levels. Responses by the innate immune system (non-specific) were measured via white blood cell counts, inflammatory cytokines and the complement system.

**Results:**

Vaccination significantly increased pneumococcal IgG levels as expected. The arabinogalactan group demonstrated a statistically significant greater IgG antibody response than the placebo group in two antibodies subtypes (18C and 23F) at both Day 51 (p = 0.006 and p = 0.002) and at Day 72 (p = 0.008 and p = 0.041). These same subtypes (18C and 23F) also demonstrated change scores from baseline which were significant, in favor of the arabinogalactan group, at Day 51 (p = 0.033 and 0.001) and at Day 72 (p = 0.012 and p = 0.003). Change scores from baseline and mean values were greater in the arabinogalactan group than placebo for most time points in antibody subtypes 4, 6B, 9V, and 19F, but these differences did not reach statistical significance. There was no effect from the vaccine or arabinogalactan on salivary IgA, white blood cell count, inflammatory cytokines or complement.

**Conclusions:**

The proprietary arabinogalactan extract (ResistAid™), tested in this randomized, double-blind, placebo-controlled, parallel-group pilot study, increased the antibody response of healthy volunteers to the 23-valent pneumococcal vaccine compared to placebo.

**Trial Registration:**

ISRCTN98817459

## Background

The immune system is a highly complex orchestration of cells, organs, tissues and active molecules which interact in an elaborate and dynamic network to protect the body from infection. The immune system can be divided into two categories: the innate immune system and the adaptive immune system. Innate immunity is an immediate but non-specific response. Adaptive or acquired immunity involves a specific reaction to a pathogen which the immune system recognizes from a previous encounter. The process of acquired immunity is the basis for vaccination[1]. Recent research has focused on the role of nutrition (foods and specific components of foods) in the responsiveness of the immune system to challenges. Vaccine-specific serum antibody production has been suggested as a highly suitable model to evaluate dietary intervention on the resistance to infection or to other immune system-related diseases[2].

The pneumococcal vaccine can reduce the incidence and/or severity of infections caused by *Streptococcus pneumoniae*: namely, pneumonia, otitis media, sinusitis and meningitis. The 23-valent vaccine contains 23 pneumococcal polysaccharide antigens (serotypes 1, 2, 3, 4, 5, 6B, 7F, 8, 9N, 9V, 10A, 11A, 12F, 14, 15B, 17F, 18C, 19A, 19F, 20, 22F, 23F and 33F[3]. Although there are at least 90 distinct serotypes, these 23 serotypes accounted for 85% to 90% of invasive pneumococcal infections in the US[3]. The 23-valent vaccine produces a humoral (antibody-mediated) response: inducing the production of antibody from B-lymphocytes in the absence of help from T-lymphocytes. The type and concentration of antibody produced is dependent on the site of exposure. Systemic administration results primarily in the generating of circulating immunoglobulin(Ig)G whereas mucosal antigenic challenge results in a more vigorous IgA response[1]. In contrast to the 23-valent pneumococcal vaccine, a 7-valent vaccine conjugated to a nontoxic diphtheria protein (used for children younger than 5 years) will induce a T-cell response[3].

Studies on improvement of the response to the pneumococcal vaccine by adults include revaccination, the addition on conjugates to the vaccine and alternative antigenic substances[4]. In addition, nutritional products have been tested on their effect on the response to vaccination. Supplementation with 200 mg/day vitamin E for 4 months to subjects at least 65 years of age caused a suggestive, but insignificant, increase in antibody response to the pneumococcal vaccine[5]. Another study evaluated the effects of prebiotic fructo-oligosaccharides (70% raftilose and 30% raftiline) derived from inulin on the response by an elderly population (70 years old and above). In this study the response to vaccination with the influenza B and pneumococcal vaccines was not significantly increased[6].

Arabinogalactans are high molecular weight, highly branched, water-soluble polysaccharides, which contain units of D-galactose and L-arabinose[7]. Arabinogalactans have previously demonstrated immunostimulatory activity[8,9]. They are present in several immune-enhancing herbs, including *Echinacea purpurea, Baptisia tinctoria, Thuja occidentalis, Angelica acutiloba, and Curucuma longa *and the medicinal mushroom *Ganoderma lucidum.*[10-12]. Arabinogalactans from Larch (*Larix *spp.) have been shown to stimulate natural killer cell cytotoxicity in vitro through the generation of interferon gamma and inhibit the metastasis of tumor cells to the liver in a rodent model[7,13,14]. A dog study demonstrated increases in white blood cell counts (due to increases in neutrophils and eosinophils), and no effect on serum IgG, IgM or IgA following oral adminstration indoses of 0.55 g/day or 1.65 g/day for 10 days[15]. A randomized, double-blind, placebo-controlled study evaluated the immunomodulating effects of a preparation of proprietary larch arabinogalactan (1.5 g/day) alone, and in combination with various preparations of Echinacea: an extract of *Echinacea purpurea *whole herb containing 4% phenolic compound (1.5 g/day), a preparation of *E. purpurea *whole herb and a preparation *E. angustifolia *root (36 to 680 mg/day)[16]. The study included 48 adult women who were divided into six groups of eight women. After 4 weeks of treatment, complement properdin increased by 18% in the group that received all four preparations and by 21% in the group given preparations of both species of Echinacea.

The current human clinical pilot study was designed to test the hypothesis that the ingestion of Resistaid™, a proprietary arabinogalactan extracted from Larch (*Larix laricina*), would selectively enhance the antibody response by adults to the 23-valent pneumococcal vaccine. Indications that the product would have immunostimulatory activity came from previous studies conducted with this proprietary product[15,16]. As there was no prior human data regarding the ability of this proprietary arabinogalactan extract to impact the immune response to the pneumococcal vaccine, a power calculation could not be performed. The sample size was set at a level consistent with prior human studies involving arabinogalactan and the immune system[16-18].

## Methods

### Investigational products

The proprietary arabinogalactan product ResistAid™, supplied by Lonza Ltd, Switzerland, contains arabinogalactan extracted from Larch (*Larix laricina*). Arabinogalactan is a highly branched polysaccharide that is composed of galactose units and arabinose units in the approximate ratio of 6:1[7]. ResistAid™ is a fine, dry, light brown powder with a neutral taste that dissolves quickly in water or juice. ResistAid™ is produced via a water extraction patented process (US 5756098; EP 86608), in accordance with Hazard Analysis and Critical Control Points (HACCP) standards and in compliance with the monograph in the Food Chemicals Codex. The Good Manufacturing Practices (GMP's) used during manufacturing are audited by the American Institute of Baking. The Larch arabinogalactan used in the ResistAid™ product has been designated as Generally Recognized as Safe (GRAS) with the US FDA (GRAS Notice Nos. GRN000047 and GRN000084).

The placebo was maltodextrin (Maltrin M100, Grain Processing Corp., USA). The test product and the placebo were administered by mixing the powders into a beverage of the subject's choice. The subjects were advised to take their dosage (4.5 g) once a day in the morning with breakfast. They began taking their assigned powder on Day 1 and continued over the entire 72 day study period.

### Subjects

Subjects between the ages of 18 and 65 were recruited for the study in the usual manner (subject database and community advertisements). Subjects were phone-screened prior to scheduling a screening visit.

Subjects were included if they were 18-65 years of age, had a Body Mass Index (BMI) ≥ 18 kg/m^2 ^and ≤ 30 kg/m^2 ^at screening, agreed to all study visits and visit procedures, agreed to use approved forms of birth control, and agreed not to initiate/change any exercise or diet programs during the study. Subjects were excluded if they had previously had the pneumococcal vaccine, had any major systemic, inflammatory or chronic disease, had any active infection or infection in the past month requiring antibiotics or anti-viral medication, used immunosuppressive drugs in the prior 5 years, were known to have alcohol or drug abuse, were pregnant or lactating or had any medical condition which in the opinion of the investigator might interfere with the subject's participation in the trial.

### Study Design

The study was a randomized, double-blind, placebo-controlled, parallel group trial with an active investigational period of 72 days. The objective was to assess the immunomodulatory effect of the arabinogalactan product on selective markers of immune function following antigenic challenge by the pneumococcal vaccine. The primary endpoints were 7 different pneumococcal IgG antibodies. The secondary objective was to determine whether the arabinogalactan product would stimulate other arms of the immune system to which there was no direct antigenic stimulus. Secondary endpoints included salivary IgA, white blood cell counts, complement (C3 and C4) and inflammatory cytokine levels. The study was conducted at the Staywell Research clinical research site located in Northridge, CA and was designed and managed by the Medicus Research Contract Research Organization (CRO) also in Northridge, CA. IRB approval was obtained prior to the initiation of any study activities (Copernicus Group IRB, Cary, NC).

Subjects meeting all of the inclusion criteria and none of the exclusion criteria for this study were randomly assigned to receive either the arabinogalactan or placebo. Double-blinding was ensured by the use of identical opaque sachets, outer packaging, labelling and color for both investigational products (arabinogalactan and placebo). Unblinding of the entire research team, including data analysis team did not occur until after the analysis was completed; subjects were blinded throughout the trial.

The study began in August 2008 (first subject in) and lasted until December 2008 (last subject completed). The subjects in the study came to the research clinic for a total of 5 visits (V1-V5) over 72 days. Subjects took the first dose of assigned study product at the screening visit (V1-Day 0) and continued to take them over the entire study. They received the 23-valent pneumococcal vaccine (Pneumovax^® ^23, Merck and Co., Inc., USA) at the vaccine visit which took place 30 days after they began taking the product or placebo (V2-Day 30). They came in for safety monitoring the day immediately following the vaccine (V3-Day 31) to observe the reaction at the vaccine administration site. Then subjects returned 21 days after vaccine (V4-Day 51) and finally 42 days after vaccine administration (V5-Day 72). On study visits, blood, urine and saliva were collected and subjects were queried regarding any change in health status. Additionally, they were assessed for compliance by interview, diary, and through the return of unused study product sachets.

The most potentially immunogenic pneumococcal antibodies (Ab) were determined in consultation with the UCLA Vaccine Center (Torrance, CA, USA) as the antibodies most likely to respond to vaccination with the 23-valent pneumococcal vaccine. These antibodies included 4, 6B, 9V, 14, 18C, 19F, and 23F. Salivary IgA was measured to monitor for non-specific effects on the adaptive immune system using immuno-array assays with a minimum sensitivity of 1.0 μg/ml. Other markers of immune function were chosen to represent the innate arm of the immune system including white blood cell counts (totals and subtypes), inflammatory cytokines, and complement (C3 and C4) determined using immuno-turbidimetric methodology. Analysis of inflammatory cytokine levels were performed using sandwich immunoassay (Affymetrix, San Diego, CA, USA). Safety monitoring included: body temperature, blood pressure, heart rate, physical exam, urinalysis, complete blood counts (CBC) and a comprehensive metabolic panel (CMP) including kidney and liver function tests.

### Analyses

Excel 2003 (Microsoft Corp, Redmond WA, USA), was used for data entry, validation, restructuring, calculating changes in variables over time, reorganizing and reformatting results, and preparing graphs. Statistical analyses were performed using SPSS Base System ver. 17 (SPSS Inc., Chicago IL, USA).

Data was analyzed using paired sample t-tests for within subject means comparisons, independent sample t-tests for between group comparisons (placebo vs. the active groups individually). Difference scores for both within and between group comparisons (placebo vs. the active groups individually) were analyzed using appropriate t-tests. Analysis was completed before the blinding code was broken.

## Results

### Subjects

Sixty five (65) subjects were screened in person at the research clinic and 53 qualified for randomization at the screening visit (V1). Of the 53, 8 did not return for V2 and therefore a total of 45 subjects were included in the intent-to-treat analysis. The subject baseline characteristics are given in Table 1.

**Table 1:** Subject Demographics

	**ResistAid**^**(™)**^	Placebo
**N**	21	24

**Male**	9 (42.9%)	16 (66.7%)

**Female**	12 (57.1%)	8 (33.3%)

**Age (range)**	33.52 (19-62)	38.25 (20-64)

### Pneumococcal IgG antibodies

Pneumococcal IgG antibody subtypes 4, 6B, 9V, 14, 18C, 19F, and 23F were measured on Days 0 (V1), 51 (V4), and 72 (V5). There were no significant differences between the groups at baseline (Day 0).

Pneumococcal IgG levels increased from baseline in response to the vaccine as expected. Supplementation with the arabinogalactan product caused a significantly greater increase from baseline in pneumococcal IgG antibody subtypes 18C and 23F at both 51 and 72 days (Table 2). Mean values between groups were also significantly greater in the arabinogalactan group for both days 51 and 72 for these two subtypes (Table 3).

**Table 2:** Effects of the 23-valent vaccine on Pneumococcal IgG antibodies

Antibody subtype	Day 0 Mean ± SD	Day 51 Mean ± SD	Day 72 Mean ± SD	Change Days 0-51	Change Days 0-72
**Type 4**	0.45 ± 0.64	2.21 ± 3.15	5.84 ± 7.35	0.023	0.042

**Type 6B**	0.95 ± 1.51	5.18 ± 6.64	5.19 ± 7.06	0.001	0.020

**Type 9V**	1.32 ± 4.10	6.07 ± 7.34	5.08 ± 5.25	0.129	0.095

**Type 14**	1.79 ± 2.56	9.91 ± 8.54	8.86 ± 8.59	0.000	0.006

**Type 18C**	0.72 ± 1.35	5.06 ± 5.80	4.93 ± 5.26	0.018	0.006

**Type 19F**	1.10 ± 2.94	7.02 ± 7.28	6.65 ± 7.26	0.011	0.015

**Type 23F**	1.08 ± 1.87	4.32 ± 4.62	4.55 ± 5.23	0.017	0.006

Increases in levels of antibody subtype as observed in the placebo group (n = 24) following inoculation with the 23-valent pneumococcal vaccine which took place on Day 30. Data are means (μg/dl) ± standard deviations on Days 0, 51 and 72. P-values for the changes between baseline and days 51 and 72 are in the right hand columns.

**Table 3:** Pneumococcal IgG types 18C and 23F - Comparisons between ResistAid^(TM) ^and Placebo Groups

	Day 0 Mean ± SD	Day 51 Mean ± SD	Day 72 Mean ± SD	Change Day 0-51	Change Day 0-72
**Type 18C**					

ResistAid™ (n = 21)	1.49 ± 3.00	9.57 ± 7.96	9.10 ± 7.53	8.08 ± 7.12	7.61 ± 6.81

Placebo (n = 24)	0.72 ± 1.35	5.06 ± 5.80	4.93 ± 5.26	4.34 ± 5.10	4.22 ± 4.69

Comparison (p-value)	0.061	0.006	0.008	0.033	0.012

**Type 23F**					

ResistAid™ (n = 21)	0.74 ± 0.93	7.07 ± 7.41	7.02 ± 7.31	6.33 ± 7.36	6.28 ± 7.17

Placebo (n = 24)	1.08 ± 1.87	4.32 ± 4.62	4.55 ± 5.23	3.24 ± 4.28	3.46 ± 4.24

Comparison (p-value)	0.059	0.002	0.041	0.001	0.003

Levels of antibody subtypes 18C and 23F in the ResistAid™ and placebo groups are given as means (μg/dl) ± standard deviations on Days 0, 51 and 72 and changes from Day 0. Inoculation with the 23-valent pneumococcal vaccine took place on Day 30. P-values are comparisons between groups and comparisons of changes from Day 0 in the two groups.

Change scores from baseline and mean values were greater in the arabinogalactan group than placebo for most time points in Ab subtypes 4, 6B, 9V, and 19F, but these differences did not reach statistical significance.

### Salivary IgA

Salivary IgA levels in the placebo group were 146 ± 109 mg/dl at baseline (Day 0). There were no significant changes from Day 0 to Days 51 or Day 0 to Day 72 in either group. There were also no significant differences in the mean values between groups.

### White blood cells

The mean total white blood cell count was 6.50 ± 1.46 × 1000/μl in the placebo group at baseline (Day 0). Comparisons between the arabinogalactan and placebo groups on Days 0, 30, 31, 51 or 72 found no significant differences in total white blood cell counts. The change from baseline Day 0 to Day 72 was significantly greater in the arabinogalactan group than the placebo group (0.38 ± 0.79 compared to 0.15 ± 1.33; p = 0.045).

Differential analysis of white blood cells determined that the levels at baseline were as follows: neutrophils 63.1 ± 5.3, lymphocytes 28.4 ± 6.0, monocytes 6.9 ± 1.9, eosinophils 1.6 ± 1.5 and basophils 0.33 ± 0.56 (measured as a percent of total white blood cells). There were no significant differences in lymphocyte, neutrophil, monocyte, or basophil counts when comparing mean values between groups at any time point. When comparing change from baseline at each time point, there were no differences between groups for lymphocytes, neutrophils, or monocytes. Change from baseline comparisons for basophils revealed a statistically significant, but clinically insignificant increase in numbers in the placebo group compared to the arabinogalactan group when comparisons were made between Day 0 and Day 72 (0.21 ± 0.72 placebo compared to 0.09 ± 0.54 arabinogalactan; p = 0.042).

Eosinophil counts were different between groups on Day 30 (2.81 ± 2.04 vs 1.46 ± 0.98; p = 0.006) and on Day 51 (3.24 ± 2.12 vs 1.83 ± 1.55; p = 0.014) with higher numbers in the arabinogalactan group. There was a larger increase in cell number (change) from baseline to Day 31 (0.14 ± 1.39 vs 0.83 ± 0.72; p = 0.035) and from baseline to Day 51 (0.48 ± 1.69 vs 0.20 ± 0.66; p = 0.006) in the arabinogalactan group.

### Complement

The levels of complement C3 and C4 at Day 0 were 125 ± 23 and 28 ± 10 mg/dl, respectively. Comparisons of means and changes from baseline for complement (C3, C4) levels between the arabinogalactan and placebo groups were not significantly different.

### Cytokines

Comparison of cytokine levels between groups found no significant differences in means for epithelial neutrophil-activating peptide (ENA)-78, eotaxin, granulocyte monocyte colony stimulating factor (GM-CSF), interferon-gamma (IFNg), interleukin (IL)-10, IL-12P40, IL-1RA, IL-2, IL-4, IL-5, IL-6, IL-8, monocyte chemotactic protein (MCP)-1, MCP-3, platelet-derived growth factor (PDGF)-BB or tumor necrosis factor (TNF)-alpha. When comparing the cytokine change from baseline values between groups, only the IL-6 change from Day 30 to Day 31 showed an increase in the arabinogalactan group compared to the placebo group. The change in the arabinoglactan group was from a mean of 17.8 ± 7.7 to 19.8 ± 7.7 pg/ml (+1.9), compared to a change from 50.1 ± 113.8 to 48.7 ± 112.5 pg/ml for the placebo group (-2.4) (p = 0.046). This was most likely in response to the vaccine which was administered on Day 30.

### Safety

No serious adverse events were reported during this study. There were nine mild adverse events in the placebo group (erythema at injection site (1), sore throat (2), nasal congestion (3), headache (2), and abdominal pain (1). There were no adverse events in the active group. All adverse events were followed by the medical staff at the research clinic.

## Discussion

The results of this pilot study suggest that the arabinogalactan preparation had a selective immunostimulating effect on acquired or adaptive immunity, as shown in the increase in antibodies without any clinically significant effects on total white blood cells, cytokines or complement. Thus it is possible that rather than acting as a general immunostimulant, arabinogalactan acted in a specific manner. The caveat to this statement is that these results are preliminary and there are confounding variables to consider.

Variables that affect the immune response to vaccines include age, gender, race and genetic characteristics[19]. One of the goals of this pilot study was to determine the effect of the intervention on a relatively broad population. As such, the study population included males and females from age 18 to 65. The randomization scheme was sequential and therefore the gender of subjects was not matched in advance. As gender and age differences may affect immunity these potentially confounding variable should be looked at in future studies. This study was an exploratory investigation into the effects of arabinogalactan with the goal of determining whether further studies are warranted. The result is that further studies with larger populations are indicated to clarify and potentially expand upon the effects of arabinogalactan on antibody production.

The suggestion that arabinogalactan might have a selective effect on the immune system is preliminary but promising. The immune system entails a complex matrix of responses to protect the body from pathogens and toxins. The innate immune system involves the rapid recruitment and upregulation of neutrophils, monocytes, macrophages, complement factors, cytokines and antimicrobial peptides to the site of infection. The innate response is the first line of host defense and the adaptive response follows a few days later. While the innate and adaptogenic arms of the immune system are often described as separate, they often act together in a synergistic manner[20]. In addition to antibodies, the variables tested in this study included salivary IgA, white blood cell counts (lymphocytes, neutrophils, monocytes, basophils and eosinophils), complement C-3 and C-4 as well as numerous cytokines. Additionally, suggestions of changes were observed in IL-6 levels and in eosinophil counts. IL-6 has immunostimulatory properties and eosinophils play a role in allergic responses. The clinical significance of these findings is unknown at this time. Additional measurements for future studies could include a breakdown of lymphocytes into subtypes, measuring natural killer (NK) lymphocytes and NK-T cells. NK cells are a heterogeneous population of innate T cells that have attracted interest because of their potential to regulate immune responses to a variety of pathogens and NK-T cells function as a bridge between innate and adaptive immunity[20].

Arabinogalactan was given for 30 days prior to vaccination and administration was continued throughout the study. The 30 days time period was chosen because a previous clinical trial studying the effect of arabinogalactan and echinacea preparations on the immune system observed a positive effect following treatment for this period of time[16].

This is the first human study to demonstrate an effect by Larch arabinogalactan on immunoglobulin levels. No effect on IgG antibody levels was observed in another study wherein the subjects were administered 1.5 g larch arabinogalactan per day for four weeks[16]. This study utilized a larger dose (4.5 g per day), longer administration time (10 weeks) and the vaccine as a standardized antigenic challenge all of which appear to have been useful in delineating a difference between the proprietary arabinogalactan extract and placebo.

## Conclusions

The proprietary arabinogalactan extract (ResistAid™) tested in this randomized, double-blind, placebo-controlled, parallel-group study, increased the antibody response of healthy volunteers to the 23-valent pneumococcal vaccine compared to placebo. The proprietary arabinogalactan product was administered safely in this study in a dose of 4.5 g per day for approximately 10 weeks. This was a pilot study that demonstrated promising effects and further studies with larger populations are indicated which may demonstrate additional effects of arabinogalactan on the immune system.

## Competing interests

The authors declare that they have no competing interests.

## Authors' contributions

JKU conceptualized the study and was the Principal Investigator. BBS also participated in the design of the study. BBS and VJS performed the analysis. JKU, BBS and MLB contributed to writing the manuscript. All authors have read and approved the final manuscript.

## References

[B1] Twigg HL III Humoral immune defense (antibodies): recent advances Proc Am Thorac Soc 2005 2 417 421 10.1513/pats.200508-089JS 16322592

[B2] Albers R Antoine JM Bourdet-Sicard R Calder PC Gleeson M Lesourd B Samartín S Sanderson IR Van Loo J Vas Dias FW Watzl B Markers to measure immunomodulation in human nutrition intervention studies Br J Nutr 2005 94 452 481 10.1079/BJN20051469 16176618

[B3] Targonski PV Poland GA Pneumococcal vaccination in adults: recommendations, trends, and prospects Cleve Clin J Med 2007 74 401 10 413 10.3949/ccjm.74.6.401 17569198

[B4] Artz AS Ershler WB Longo DL Pneumococcal vaccination and revaccination of older adults Clin Microbiol Rev 2003 16 308 318 10.1128/CMR.16.2.308-318.2003 12692100 PMC153142

[B5] Meydani SN Meydani M Blumberg JB Leka LS Siber G Loszewski R Thompson C Pedrosa MC Diamond RD Stollar BD Vitamin E supplementation and in vivo immune response in healthy elderly subjects. A randomized controlled trial JAMA 1997 277 1380 1386 10.1001/jama.277.17.1380 9134944

[B6] Bunout D Hirsch S Pia dlM Munoz C Haschke F Steenhout P Klassen P Barrera G Gattas V Petermann M Effects of prebiotics on the immune response to vaccination in the elderly JPEN J Parenter Enteral Nutr 2002 26 372 376 10.1177/0148607102026006372 12405649

[B7] Kelly GS Larch arabinogalactan: clinical relevance of a novel immune-enhancing polysaccharide Altern Med Rev 1999 4 96 103 10231609

[B8] Beuth J Ko HL Oette K Pulverer G Roszkowski K Uhlenbruck G Inhibition of liver metastasis in mice by blocking hepatocyte lectins with arabinogalactan infusions and D-galactose J Cancer Res Clin Oncol 1987 113 51 55 10.1007/BF00389966 3818778 PMC12248396

[B9] Beuth J Ko HL Schirrmacher V Uhlenbruck G Pulverer G Inhibition of liver tumor cell colonization in two animal tumor models by lectin blocking with D-galactose or arabinogalactan Clin Exp Metastasis 1988 6 115 120 10.1007/BF01784842 3345610

[B10] Roxas M Jurenka J Colds and influenza: a review of diagnosis and conventional, botanical, and nutritional considerations Altern Med Rev 2007 12 25 48 17397266

[B11] Classen B Thude S Blaschek W Wack M Bodinet C Immunomodulatory effects of arabinogalactan-proteins from Baptisia and Echinacea Phytomedicine 2006 13 688 694 10.1016/j.phymed.2005.10.004 17085292

[B12] Luettig B Steinmuller C Gifford GE Wagner H Lohmann-Matthes ML Macrophage activation by the polysaccharide arabinogalactan isolated from plant cell cultures of Echinacea purpurea J Natl Cancer Inst 1989 81 669 675 10.1093/jnci/81.9.669 2785214

[B13] Currier NL Lejtenyi D Miller SC Effect over time of in-vivo administration of the polysaccharide arabinogalactan on immune and hemopoietic cell lineages in murine spleen and bone marrow Phytomedicine 2003 10 145 153 10.1078/094471103321659852 12725568

[B14] D'Adamo P Larch Arabinogalactan is a Novel Immune Modulator J Naturopath Med 1996 4 32 39

[B15] Grieshop CM Flickinger EA Fahey GC Jr Oral administration of arabinogalactan affects immune status and fecal microbial populations in dogs J Nutr 2002 132 478 482 11880574 10.1093/jn/132.3.478

[B16] Kim LS Waters RF Burkholder PM Immunological activity of larch arabinogalactan and Echinacea: a preliminary, randomized, double-blind, placebo-controlled trial Altern Med Rev 2002 7 138 149 11991793

[B17] Nantz M Painter A Parker E McGill C Percival S Evaluation of arabinogalactan's effect on human immunity FASEB J 2001 15 A633

[B18] Causey J Robinson R Feirtag J Fulcher R Slavin J Effects of larch arabinogalactan on human peripheral blood mononuclear cells: results from in vivo and in vitro human trials FASEB J 1999 13 A589

[B19] Thomas C Moridani M Interindividual variations in the efficacy and toxicity of vaccines Toxicology 2009 in press 19837123 10.1016/j.tox.2009.10.008

[B20] Chaplin DD Overview of the immune response J Allergy Clin Immunol 2010 125 S3 23 10.1016/j.jaci.2009.12.980 20176265 PMC2923430

